# Unobtrusive Sleep Posture Detection Using a Smart Bed Mattress with Optimally Distributed Triaxial Accelerometer Array and Parallel Convolutional Spatiotemporal Network

**DOI:** 10.3390/s25123609

**Published:** 2025-06-08

**Authors:** Zhuofu Liu, Gaohan Li, Chuanyi Wang, Vincenzo Cascioli, Peter W. McCarthy

**Affiliations:** 1 The Higher Educational Key Laboratory for Measuring and Control Technology and Instrumentations of Heilongjiang Province, Harbin University of Science and Technology, Harbin 150080, China; 2 Murdoch University Chiropractic Clinic, Murdoch University, Murdoch 6150, Australia; cascioli.family@googlemail.com; 3 Faculty of Life Science and Education, University of South Wales, Treforest, Pontypridd CF37 1DL, UK; peter.mccarthy@southwales.ac.uk; 4 Faculty of Health Sciences, Durban University of Technology, Durban 1334, South Africa

**Keywords:** sleep posture classification, non-contact detection, deep learning module, density peak clustering algorithm, triaxial accelerometers, parallel convolutional spatiotemporal networks

## Abstract

Sleep posture detection is a potentially important component of sleep quality assessment and health monitoring. Accurate identification of sleep postures can offer valuable insights into an individual’s sleep patterns, comfort levels, and potential health risks. For example, improper sleep postures may lead to musculoskeletal issues, respiratory disturbances, and even worsen conditions like sleep apnea. Additionally, for long-term bedridden patients, continuous monitoring of sleep postures is essential to prevent pressure ulcers and other complications. Traditional methods for sleep posture detection have several limitations: wearable sensors can disrupt natural sleep and cause discomfort, camera-based systems raise privacy concerns and are sensitive to environmental conditions, and pressure-sensing mats are often complex and costly. To address these issues, we have developed a low-cost non-contact sleeping posture detection system. Our system features eight optimally distributed triaxial accelerometers, providing a comfortable and non-contact front-end data acquisition unit. For sleep posture classification, we employ an improved density peak clustering algorithm that incorporates the K-nearest neighbor mechanism. Additionally, we have constructed a Parallel Convolutional Spatiotemporal Network (PCSN) by integrating Convolutional Neural Network (CNN), Long Short-Term Memory (LSTM), and Bidirectional Long Short-Term Memory (Bi-LSTM) modules. Experimental results demonstrate that the PCSN can accurately distinguish six sleep postures: prone, supine, left log, left fetus, right log, and right fetus. The average accuracy is 98.42%, outperforming most state-of-the-art deep learning models. The PCSN achieves the highest scores across all metrics: 98.64% precision, 98.18% recall, and 98.10% F1 score. The proposed system shows considerable promise in various applications, including sleep studies and the prevention of diseases like pressure ulcers and sleep apnea.

## 1. Introduction

Sleep posture detection is a crucial aspect of sleep research and clinical practice, as it can provide valuable insights into sleep quality and the prevention of various health issues [1]. Sleep posture can affect a wide range of physiological processes, including respiration, spinal alignment, and overall comfort during sleep. For instance, improper sleep postures may lead to tension in the cervical and lumbar spine, causing pain, and potentially contributing to related diseases such as obesity, diabetes mellitus, and hypertension [2,3,4]. Additionally, inadequate sleep or sleep disturbances have been linked to an increased risk of depression, anxiety, and various other mental or psychiatric disorders [5]. The prevalence of sleep disorders is estimated to be 4.7% in the population, with some reports indicating that up to half of the population may experience sleep-related disorders in Western countries [6].

With the global elderly population rapidly increasing and projected to account for 20% of the total world population by 2050 [7], obtaining adequate and restful sleep is particularly important for this demographic. Sleep enables the body and brain to undergo essential restorative processes necessary for overall health and cognitive function recovery [8,9]. Studies have shown that sleep-related issues are more prevalent among individuals in residential care settings [10], with falling out of bed during sleep representing a significant risk for the elderly, potentially leading to injuries and, in severe cases, even death [11]. Moreover, poor sleep postures are considered major contributors to certain health complications, especially among the elderly, including impaired physical health, cognitive decline, and a reduced overall quality of life [12], which is especially important among the elderly. Additionally, adjusting postures in cardiovascular patients has been shown to help reduce cardiac load [13,14,15,16].

In the medical field, long-term bedridden patients who maintain the same lying posture for extended periods are at an increased risk of developing pressure sores/ulcers [1,5,6], with body position detection being considered essential for preventing the formation of pressure sores. Therefore, sleep posture detection could prove crucial for alerting individuals to avoid improper positions, or prolonged periods of any position, and for reminding people to change posture regularly, thereby reducing the risk of any associated health complications [17].

The objective of this study is to explore the feasibility of accurately identifying sleep postures using low-complexity hardware configurations. To accomplish this, we have developed a smart mattress equipped with eight triaxial accelerometers, whose output data is analyzed using a Parallel Convolutional Spatiotemporal Network (PCSN) model to enable highly precise sleep posture recognition. The main contributions of this work include: (1) the design of a cost-effective smart mattress with minimal sensor requirements, (2) the development of a novel PCSN model specifically optimized for sleep posture recognition, and (3) a comprehensive evaluation demonstrating the system’s high accuracy in real-world scenarios.

In the following sections, we first summarize the achievements of state-of-the-art publications in Section 2. We then detail the unobtrusive sleep posture detection system, including accelerometer evaluation, optimal distribution of the eight accelerometers, and related algorithms in Section 3. In Section 4, we explore hyperparameter selection, system accuracy, and classification performance from both metrological and model metrics perspectives. In Section 5, we compare the proposed system against classic non-contact sleep posture detection methods and discuss the limitations of the current study. Finally, we provide a conclusion in Section 6.

## 2. Related Works

Traditional methods for sleep posture detection can be broadly categorized into four main approaches: wearable device-based [18,19], camera-based [20,21], radio-frequency-based [22], and pressure-sensitive mat-based [23,24,25,26]. Wearable devices, such as accelerometers and gyroscopes, are attached directly to the human body to monitor movements and postures. Camera-based systems use video or infrared cameras to capture images and analyze sleep postures. Radio-frequency-based methods detect sleep postures through the reflection of radio waves. Pressure-sensitive mats, in contrast, measure the pressure distribution of the body to infer sleep postures.

However, these traditional methods have several drawbacks. Wearable devices can cause discomfort and interfere with normal sleep. Camera-based systems may raise privacy concerns and are highly dependent on ambient lighting. Radio-frequency-based methods can be expensive, require complex installation, and are susceptible to multipath effects and environmental interference [22]. Pressure-sensitive mats, although more comfortable and less invasive, often involve large-scale sensor arrays that increase production costs and computational complexity. Additionally, these mats may suffer from area redundancy, as only a portion of the sensors are utilized when a person lies on the mat. These limitations highlight the need for a more efficient, cost-effective, and non-contact method to detect different sleep postures [27,28].

In recent years, significant progress has been made in sleep posture classification driven by deep learning techniques. For example, Andy Yiu-Chau Tam et al. [20] proposed a deep learning model integrated with an ECA (Efficient Channel Attention) mechanism, which improved the F1 score from 87.4% to 92.2%. While this method significantly enhanced classification accuracy, its performance was limited in low-light environments due to its reliance on RGB images. In another study, Zebo Li et al. [29] introduced a dual-fusion recognition model based on an air pressure cushion. This model combines human anatomical features with algorithmic enhancements, achieving an accuracy of 99.9%. Experimental results indicated that the method performed exceptionally well on low-density air cushion data. However, given that the entire system comprises 264 nodes, further structural optimization is necessary to enhance its efficiency and effectiveness. Despite these advancements in signal processing and algorithmic structures, challenges remain in terms of environmental adaptability, cost-effectiveness, non-invasiveness, and practical deployment.

In contrast to existing approaches, our work advances the state-of-the-art by proposing a systematic structure that addresses key limitations of traditional methods. Our smart mattress, equipped with only eight triaxial accelerometers, significantly reduces hardware complexity compared to existing pressure-sensitive mat systems with hundreds of sensors. Furthermore, our PCSN model achieves highly accurate sleep posture recognition while maintaining computational efficiency. This approach balances non-invasiveness, cost-effectiveness, and performance, making it particularly suitable for practical deployment in both clinical and home settings.

## 3. Materials and Methods

While manufacturers perform batch sampling on available sensors, it is crucial to assess their specifications, including consistency and linearity. This is especially important when multiple sensors, such as triaxial accelerometers in this study, are used together for data collection.

### 3.1. Accelerometer Performance Evaluation

**Figure 1:**
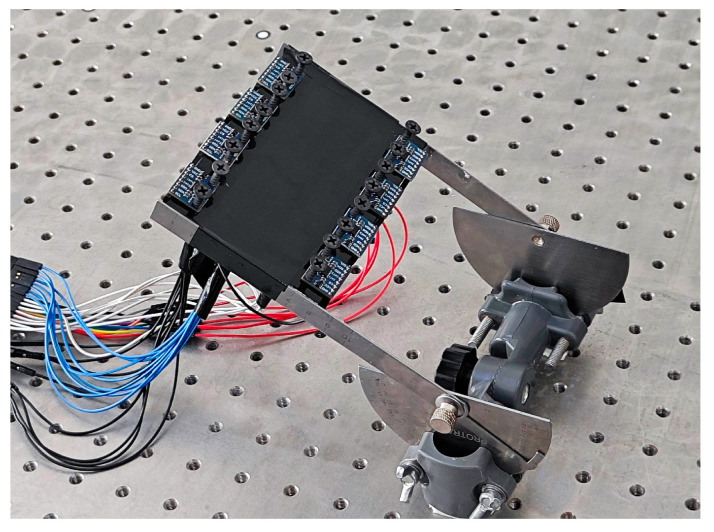
Performance evaluation of eight triaxial accelerometers using a customized rotating device installed on a high-precision optical platform.

To assess the triaxial accelerometer (ADXL345, Analog Devices Inc., Wilmington, MA, USA), we employed a custom-designed rotating device [30] capable of rotating through a 180° arc. This device was mounted on an optical platform to enhance the stability of the evaluation system. Eight triaxial accelerometers were positioned along the rotating device (Figure 1), with each axis aligned sequentially. The alignment process began with the x-axis, followed by the y-axis, and finally the z-axis. For each axis alignment, the rotating device with the triaxial accelerometers was turned in 5° increments. The sampling frequency was set to 240 Hz. The selection of a 240 Hz sampling frequency is based on the requirements of the eight-channel accelerometer data acquisition in our system. Specifically, this sampling rate ensures that each channel operates at a frequency of 30 Hz. This frequency is within the range of 13 to 35 Hz, which is deemed sufficient for effective human motion detection [18,31].

### 3.2. Optimal Placement of Accelerometers

**Figure 2:**
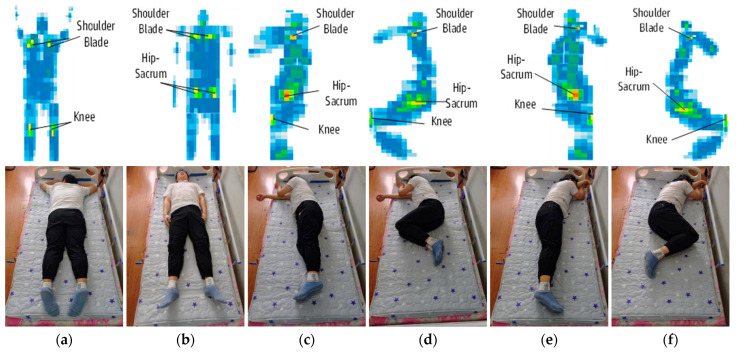
Six sleep positions to be classified in the experiment and corresponding pressure distribution maps. The colors in the heat map represent the relative pressure levels, with a gradient indicating increasing load intensity. Specifically, light blue denotes the lowest pressure, followed by dark blue, green, yellow, and red, which signifies the highest pressure. Top row shows a heat map of pressure distribution for each posture, with warmer colors (yellow–red) indicating higher pressure points at key anatomical landmarks (scapula, hip-sacrum, and knee). Bottom row shows corresponding postures captured by cameras: (**a**) prone—face down with arms on either side of the body; (**b**) supine—face up with arms on either side of the body; (**c**) left log—lying on the left side with the straight legs; (**d**) left fetus—lying on the left side with bent legs; (**e**) right log—lying on the right side with straight legs; (**f**) right fetus—lying on the right side with bent legs.

To determine the optimal placement of accelerometers under the mattress, preliminary tests were conducted for six typical sleep postures: prone, supine, left log, left fetus, right log, and right fetus [32]. These tests involved placing a force-sensitive mat under the body of a volunteer (Figure 2). The points with the highest pressure were then selected as the locations for the triaxial accelerometers. The results of the preliminary tests show that the primary stress points are in the shoulder blade, spine, coccyx, hip-sacrum, and knees.

**Figure 3:**
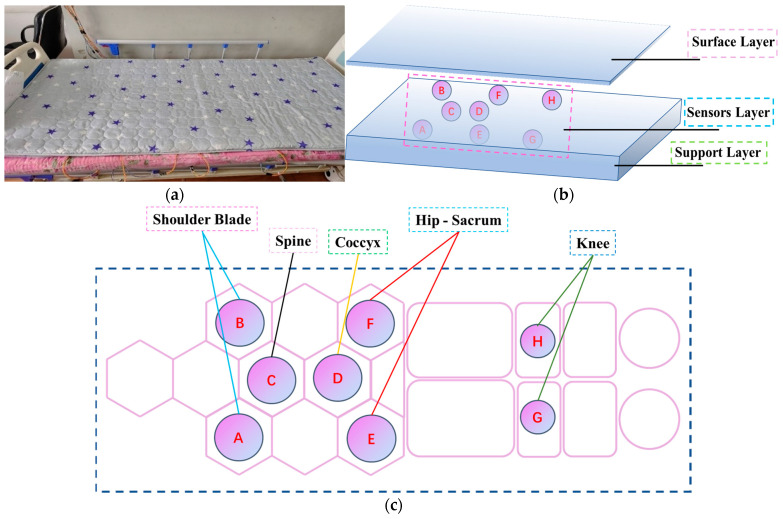
Sleep detection mattress. (**a**) Photo of experimental mattress embedded with eight triaxial accelerometers; (**b**) three-layer structure of smart mattress: top layer is commercially available mattress, middle layer contains an optimally distributed triaxial accelerometer array (labeled A–H), and bottom layer provides structural support; (**c**) top view of optimally distributed accelerometer array. Accelerometers are placed at key anatomical pressure points: shoulder blade (accelerometer A and B), spine (accelerometer C), coccyx (accelerometer D), hip-sacrum (accelerometer E and F), and knees (accelerometer G and H). Optimally placed accelerometers reduce system complexity by focusing on key pressure points identified by preliminary trials (Figure 2).

By identifying sensitive areas, eight triaxial accelerometers were placed around these regions to optimally cover the key features of various sleep postures. This layout minimizes system complexity by optimizing the spatial resolution of the sensors, thereby providing an effective hardware configuration for the subsequent development of the sleep posture detection system. A 190 cm × 90 cm mattress was then placed on top of the optimized accelerometer array for sleep posture detection (Figure 3).

It should be noted that while our sensor placement strategy was derived from tests with a volunteer of average body type (height: 170.7 cm, weight: 61.8 kg), the identified pressure points generally correspond to anatomical landmarks that are common across different body types. The relative positions of these landmarks may vary with individual height and weight, but the primary stress points (shoulder blade, spine, coccyx, hip-sacrum, and knees) remain consistent across most body compositions. The system proposed in this study relies on the pattern of pressure distribution rather than absolute pressure values, which provides some inherent adaptability to different body types. However, extreme variations in height (below 160 cm or above 190 cm) or weight may affect the system’s accuracy, as the pressure distribution patterns could significantly deviate from those observed in our preliminary tests. This limitation is further addressed in Section 4.3, where we discuss potential future work to enhance the system’s adaptability and applicability across diverse populations.

The system employs an FPGA-based architecture (EP4CE10F17C8N, Intel Corpo-ration, Santa Clara, CA, USA) to interface with eight accelerometers via dedicated I^2^C connections. Each triaxial accelerometer is assigned an independent pair of SCL and SDA lines, enabling parallel and isolated communication. Eight I^2^C driver modules are implemented to handle sensor initialization, register configuration, and data acquisition. The output from each of triaxial accelerometers is a 48-bit data package (data [47:0]), representing 16-bit acceleration values along the *x*, *y*, and *z* axes, respectively.

Within the FPGA, a polling mechanism sequentially accesses each triaxial accelerometer and forwards the acquired data to a processing unit. This unit formats the data with the assistance of clock division and state machine control modules. The formatted data is then transmitted via a Universal Asynchronous Receiver/Transmitter (UART) interface to a laptop at a baud rate of 115,200 bits per second.

### 3.3. Participants

Participants in the study were healthy adults aged 18 years or older. Individuals who suffered from illnesses such as sleep deprivation, sleep disturbances, or musculoskeletal pain, or who had difficulties maintaining or transitioning between specific postures in bed, were excluded. Before the trials, all participants were requested to not engage in intense walking or running activities. The experiment was approved by the Faculty Research Committee (No. 20241118) and conducted in accordance with the guidelines outlined in the Declaration of Helsinki. A total of 30 healthy university students (15 males and 15 females) participated in the experiment. All participants provided informed consent after receiving a verbal and written description of the experimental procedures. Basic demographic information for the participants is presented in Table 1.

### 3.4. Experimental Procedures

The trials were conducted from 20 November to 10 December 2024. During the experiment, each subject lay on the mattress embedded with eight triaxial accelerometers underneath and assumed six distinct sleeping positions (Figure 1) randomly to avoid potential order effects. While maintaining the fundamental posture of each sleeping position, minor positional adjustments were made to enhance the diversity of the data collected for each posture. Every participant remained in a given sleep posture for at least 30 s before transitioning to the next, thereby ensuring the acquisition of no fewer than 25 sets of acceleration data per posture. Consequently, a total of 4500 data points were obtained for all six sleep postures (25 × 6 × 30 = 4500). All the recorded data were organized into a sleep posture dataset for subsequent analysis and processing.

### 3.5. Statistical Analysis for Accelerometer Performance

The data were analyzed using Python (version 3.8.10) and Excel 2021 (Microsoft, Seattle, USA). One-way ANOVA was employed to access the consistency within the datasets gathered from the triaxial accelerometers. A significance level of <0.05 was considered indicative of statistical significance.

### 3.6. Data Preprocessing with Improved Density Peak Clustering

Clustering, as an unsupervised learning method, aims to uncover the underlying structure of unlabeled data by exploring similarity relationships in feature space [33]. Among various clustering algorithms, such as K-means [34], hierarchical clustering [35], and self-organizing maps (SOM) [36], density peak clustering (DPC) [37] stands out for its ability to identify clusters based on local density and distance. However, in this study, the distribution of sleep posture samples exhibited notable inconsistency due to individual variability in height and weight among participants. Traditional DPC struggles with such heterogeneity because of its dependence on a global cutoff distance for density estimation, leading to three main limitations:

1. Difficulty in distinguishing intra-class differences and inter-class similarities;
2. Inability to automatically identify cluster centers;
3. Increased subjectivity due to human intervention.

To address these issues, we proposed an improved clustering framework that integrates the K-nearest neighbor (KNN) strategy with the DPC algorithm. This approach reconstructs key components of the traditional DPC pipeline by improving adaptability and robustness.

1. Local Density

Instead of using a global truncation distance, we redefine the local density based on the average distance between a sample point $x_{\alpha }$ and its *k*-nearest neighbors:

(1) $$\rho_{\alpha }=1-\frac{\left(\sum_{x_{\beta }\in KNN(x_{\alpha }),x_{\beta }\neq x_{\alpha }}d(x_{\alpha },x_{\beta })\sigma^{-1}\right)}{k}$$

where $d(x_{\alpha },x_{\beta })$ denotes the Euclidean distance between two sample points, and $\sigma =\max\{d(x_{\alpha },x_{\beta }),x_{\alpha },x_{\beta }\in X$} serves as a normalization factor. This formulation effectively mitigates the sparsity effect in high-dimensional space and reflects how densely $x_{\alpha }$ is surrounded by other points. The default value for *k* is 8.

2. Repulsion Factor

To distinguish common points (points located in areas with relatively uniform density) from central points (points located at the center of high-density areas) or isolated points (points located in areas with lower density), a repulsion factor $\delta_{a}$ is used:

(2) $$\delta_{\alpha }=\left\{\begin{array}{ll} \underset{x_{\beta }\in KNN\prime (x_{\alpha }),x_{\varepsilon }\in HD(x_{i})}{\min}d(x_{\beta },x_{\varepsilon }) & if\;\rho (x_{\alpha })<\underset{x_{\phi }\in X}{\max\rho (x_{\phi })}\\ \underset{x_{\beta }\in X}{\max}\;d(x_{\beta },x_{\varepsilon }) & otherwise \end{array}\right.$$

where ${KNN}^{\prime }(x_{\alpha })=KNN(x_{\alpha })\cup x_{\alpha }$, $HD(x_{\alpha })=\left\{x_{\varepsilon }\right.|\rho_{\varepsilon }>\rho_{\alpha },x_{\varepsilon }\in \left.X\right\},\;x_{\emptyset }$ is the sample point in the sample set X. This metric reflects the proximity of $x_{\alpha }$ to a higher-density region. If $x_{\alpha }$ is a peak point, the global maximum distance is used.

3. Centrality Evaluation

The overall importance of a point is evaluated by combining its local density and repulsion factor values. The centrality score is defined as:

(3) $$\gamma_{\alpha }={\left(\rho_{\alpha }\times \delta_{\alpha }\right)}^{m}$$

where the default value for *m* is 2.

4. Adaptive Center Selection

Data points whose local density and repulsion factor values exceeding the average levels are considered as the potential cluster centers:

(4) $$Centers=\{x_{\alpha }|\rho_{\alpha }>\bar{\rho }\cap \;\delta_{\alpha }>\bar{\delta }\}$$

where $\bar{\rho }$ and $\bar{\delta }$ represent the mean values of local density and repulsion factor value, respectively.

5. Point gap

After identifying the candidate cluster centers, all candidates are sorted in descending order according to their centrality scores $\gamma_{\alpha }$ as defined in Equation (3). To determine the optimal number of clusters, the difference between each pair of adjacent centrality values is:

(5) $$g_{\alpha }=\gamma_{\alpha }-\gamma_{\alpha +1}$$

The average difference $\bar{g}$ is computed over all $g_{\alpha }$ values. The index of the first occurrence where $g_{\alpha }>\bar{g}$ serves as the estimated number of clusters. For instance, if this condition is first met at $g_{3}=\gamma_{3}-\gamma_{4}$, then the top three candidate points (i.e. $\gamma_{1},\;\gamma_{2},\;\gamma_{3}$) are selected as the final cluster centers. This strategy ensures that only points with relatively high centrality are preserved, while suppressing outliers or ambiguous candidates. The pseudocode of the improved DPC algorithm is included in the Appendix A (Algorithm A1).

### 3.7. Model Training and Architecture

**Figure 4:**
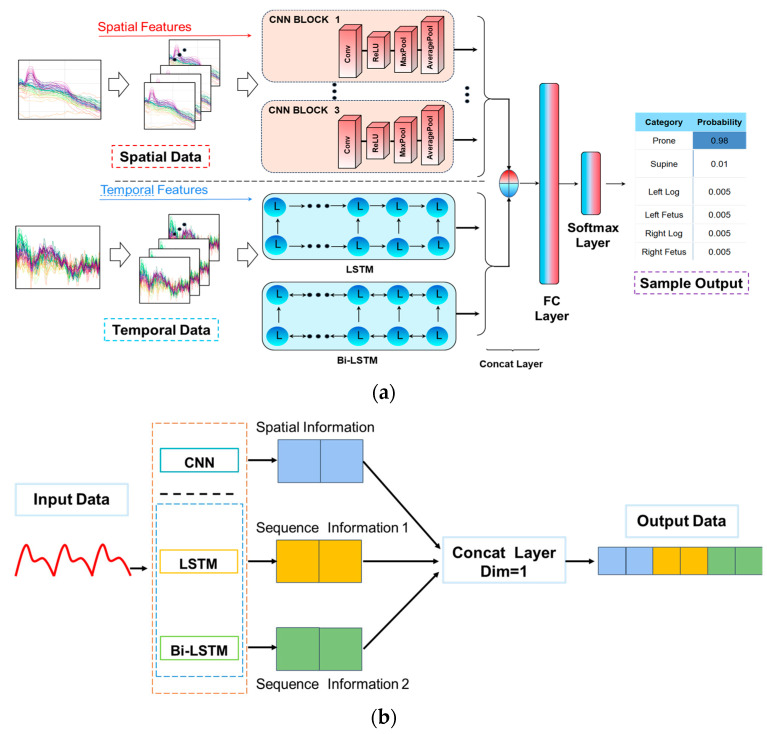
Structure of proposed PCSN model: (**a**) overall network architecture; (**b**) concatenated feature fusion module.

To address the limitations of traditional serial CNN and LSTM architectures, where temporal dependencies are often weakened due to feature loss during convolution and pooling [38,39], we propose a novel Parallel Convolutional Spatiotemporal Network (PCSN). As illustrated in Figure 4a, PCSN employs a dual-path design to simultaneously extract spatial and temporal features, thereby enhancing classification performance on sleep posture data.

The upper branch of the PCSN model comprises three parallel CNN blocks. Each block includes a stack of convolutional layers, ReLU activations, max pooling, and average pooling. These blocks capture spatial correlation patterns among multi-channel accelerometer signals and enhance discriminability in feature space.

In the lower branch, temporal features are extracted using both LSTM and Bi-LSTM (Bidirectional LSTM) modules.

- LSTM [40] captures long-term dependencies within time domain while alleviating gradient vanishing.
- Bi-LSTM [41] enhances temporal awareness by processing data bidirectionally—both forward and backward—enabling better understanding of contextual relationships across time.

The features from CNN, LSTM, and Bi-LSTM modules are concatenated along the feature dimension, as illustrated in Figure 4b. This concatenated fusion [42] combines spatial and temporal representations into a unified feature vector, yielding a richer and more comprehensive data representation. Each module contributes information in distinct dimensions, and the fusion process preserves both position information (spatial) and sequential cues (temporal) essential for sleep posture classification.

The fused feature vector is passed into a fully connected (FC) layer, which maps the high-dimensional features to the final label space. A Softmax activation function is applied at the output layer to generate a probability distribution across predefined categories (e.g., prone, supine, left log, etc.). The sleep posture category corresponding to the highest probability is selected as the final prediction result.

### 3.8. Performance Analysis Tools for Improved DPC

NMI (Normalized Mutual Information) [43] and ARI (Adjusted Rand Index) [44] are used to evaluate the clustering performance. NMI is an indicator used to measure the similarity between clustering results and true labels, with a value range of [0, 1]. When the value is closer to 1, it means that the clustering result is closer to the true category. Its definition is:

(6) $$NMI(U,V)=\frac{2\cdot I(U,V)}{H\left(U\right)+H(V)}$$

where U is the true classification, *V* is obtained by the clustering algorithm, and *I*(U,*V*) is the mutual information, which represents the common information of the two distributions:

(7) $$I(U,V)=\sum_{i}\sum_{j}p(i,j)log\frac{p(i,j)}{p(i)p(j)}$$

where *p*(.) is defined as the probability of the corresponding variable, while *H*(U) and *H*(*V*) represent entropy:

(8) $$H(\;U\;)=-\sum_{i}p\left(i\right)logp(i)$$

(9) $$H(V)=-\sum_{i}p\left(j\right)logp(j)$$

ARI is an improved version of the rand index and is used to measure the consistency between clustering results and true classification [45]. It takes into account the impact of random clustering and makes adjustments to avoid high scores caused by randomness. When the value is close to 1, it means that the clustering results are very consistent with the true category; when the value is close to 0, it means that the clustering results are close to random.

(10) $$ARI=\left|\frac{\sum_{ij}\left(\begin{array}{c} n_{ij}\\ 2 \end{array}\right)-\left[\sum_{i}\left(\begin{array}{c} a_{i}\\ 2 \end{array}\right)\sum_{j}\left(\begin{array}{c} b_{j}\\ 2 \end{array}\right)/\left(\begin{array}{c} n\\ 2 \end{array}\right)\right]}{\frac{1}{2}\left[\sum_{i}\left(\begin{array}{c} a_{i}\\ 2 \end{array}\right)+\sum_{j}\left(\begin{array}{c} b_{j}\\ 2 \end{array}\right)\right]-\left[\sum_{i}\left(\begin{array}{c} a_{i}\\ 2 \end{array}\right)\sum_{j}\left(\begin{array}{c} b_{j}\\ 2 \end{array}\right)/\left(\begin{array}{c} n\\ 2 \end{array}\right)\right]}\right|$$

where $n_{ij}$ represents the number of data points that belong to both cluster *i* in the true classification and cluster *j* in the clustering result. $a_{i}$ denotes the total number of data points in cluster *i* of the true classification, while $b_{j}$ denotes the total number of data points in cluster *j* of the clustering result. $\left(\begin{array}{c} n\\ 2 \end{array}\right)$ represents the number of possible pairs that can be formed by choosing any two samples from a dataset of size *n*. This combinatorial expression is used to calculate the total number of sample pairs for evaluating clustering consistency.

## 4. Results

### 4.1. Experimental Environment and Hyperparameter Selection

In the training process of deep learning models, processing large amounts of data requires significant computational resources. To meet this demand, we utilized a GPU to train the PCSN model. Specifically, an NVIDIA GeForce RTX 4060 graphics card was employed, in conjunction with the CUDA parallel computing architecture and the cuDNN acceleration library. To ensure efficient training, the PyTorch (version 1.7.1+cu101) framework was selected for this experiment, as it effectively supports the implementation and optimization of complex algorithms.

**Figure 5:**
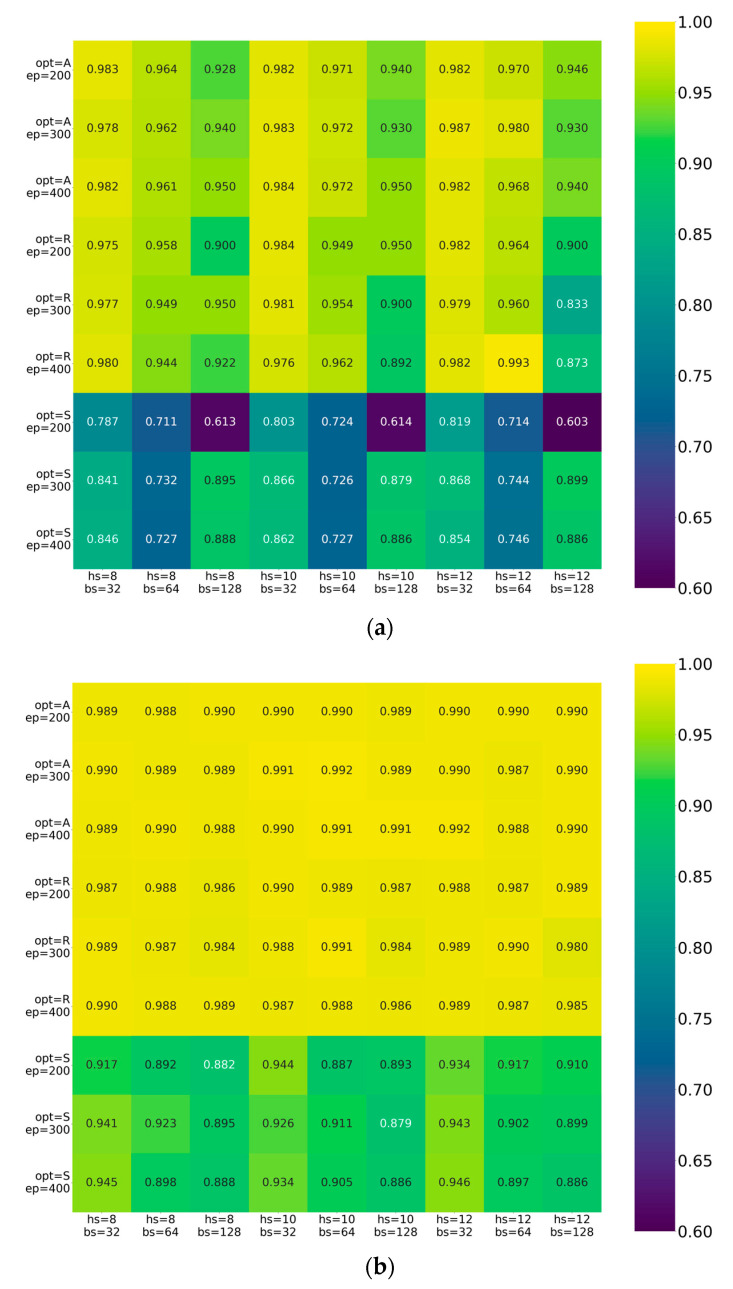

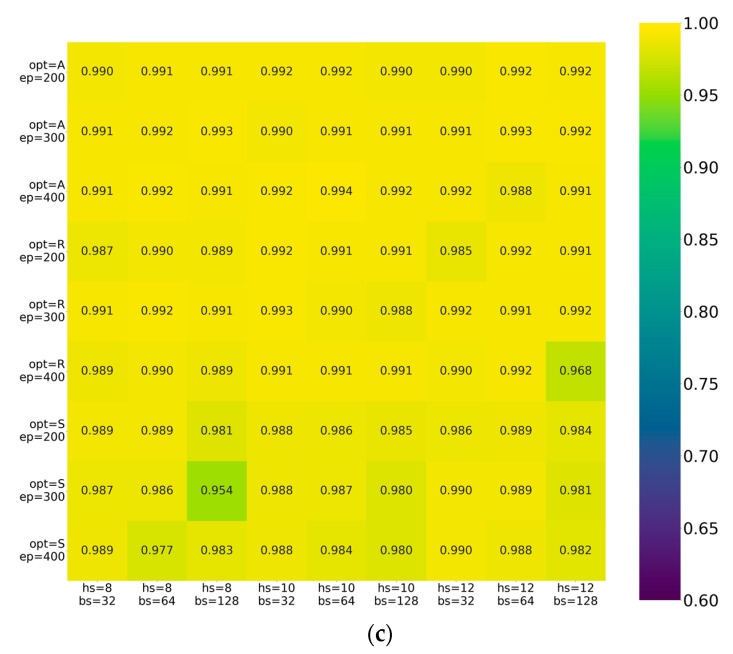
Comparison results of grid search and cross validation for selecting optimal hyperparameters, including learning rate (lr), batch size (bs), epochs (ep), optimizer (opt), and hidden size (hs). (**a**) lr = 0.01, (**b**) lr = 0.001, (**c**) lr = 0.0001.

To determine the optimal hyperparameters for the PCSN model, we conducted a prior selection test using the grid search approach and cross-validation scheme. The model was implemented using the PyTorch framework, and the comparison results were partially illustrated (Figure 5) due to too many combinations in hyperparameters (663 combinations in total). The heat map analysis further reveals the complex relationships between the hyperparameters. The optimal hyperparameter settings included the Adam optimizer, learning rate of 0.0001, batch size of 64 samples, hidden size of 10, and a total of 400 epochs to ensure convergence. The optimal combination performs well not only in each individual dimension but also maintains strong correlations after accounting for parameter interactions.

### 4.2. Performance of Accelerometer

**Figure 6:**
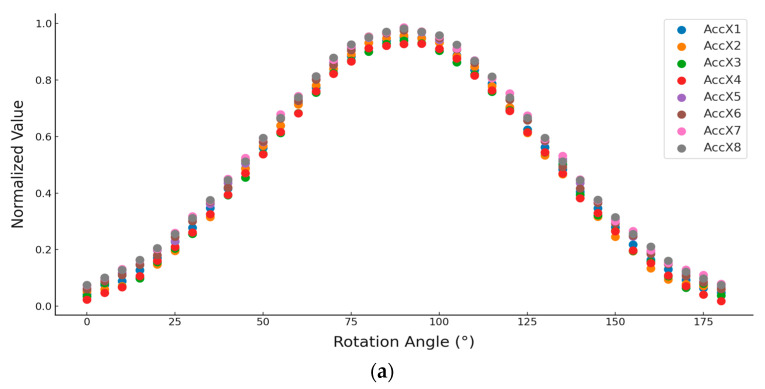

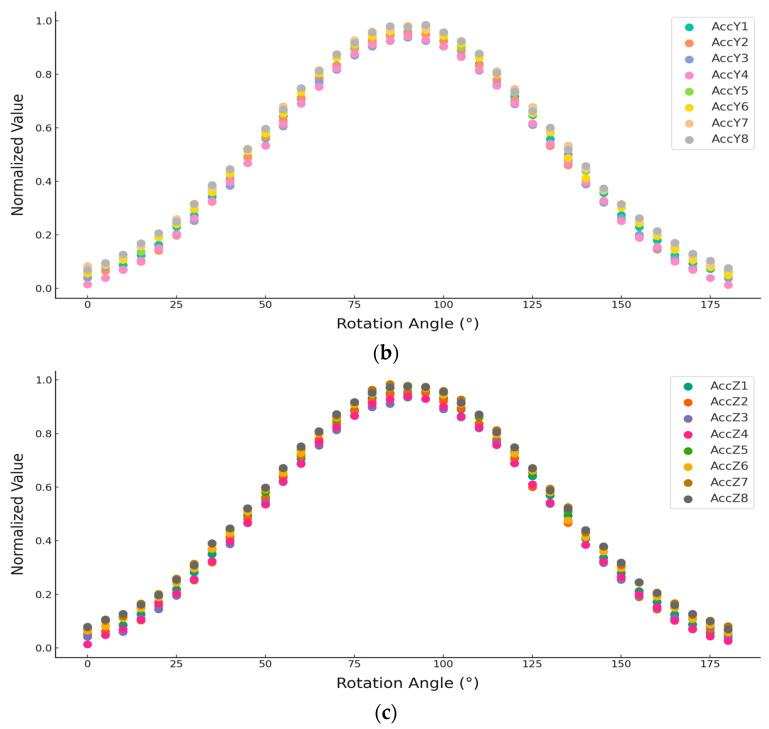
Response characteristic curve of eight triaxial accelerometers with a sampling interval of 5° in range of 0° to 180° using custom-made rotating device. (**a**) *x*-axis data, (**b**) *y*-axis data, (**c**) *z*-axis data.

The ANOVA was conducted on each axis of the eight triaxial accelerometers, indicating no significant difference (*p* > 0.05). Moreover, the curves (Figure 6) demonstrated strong consistency as the sensors were rotated from 0° to 180°.

### 4.3. System Performance from a Metrological Point of View

The entire system comprises three main components: (1) a bed frame, which is based on a standard hospital bed frame and provides structural support for the entire system; (2) a sensor layer, which includes embedded triaxial accelerometers positioned optimally at corresponding body pressure points; and (3) a mattress, which offers comfort to users while effectively transmitting body movements to the accelerometers below. To evaluate the system performance from a metrological perspective, we present three key factors: system accuracy, spatial resolution, and acceleration resolution across three axes (Table 2).

### 4.4. Comparative Test for Clustering

**Figure 7:**
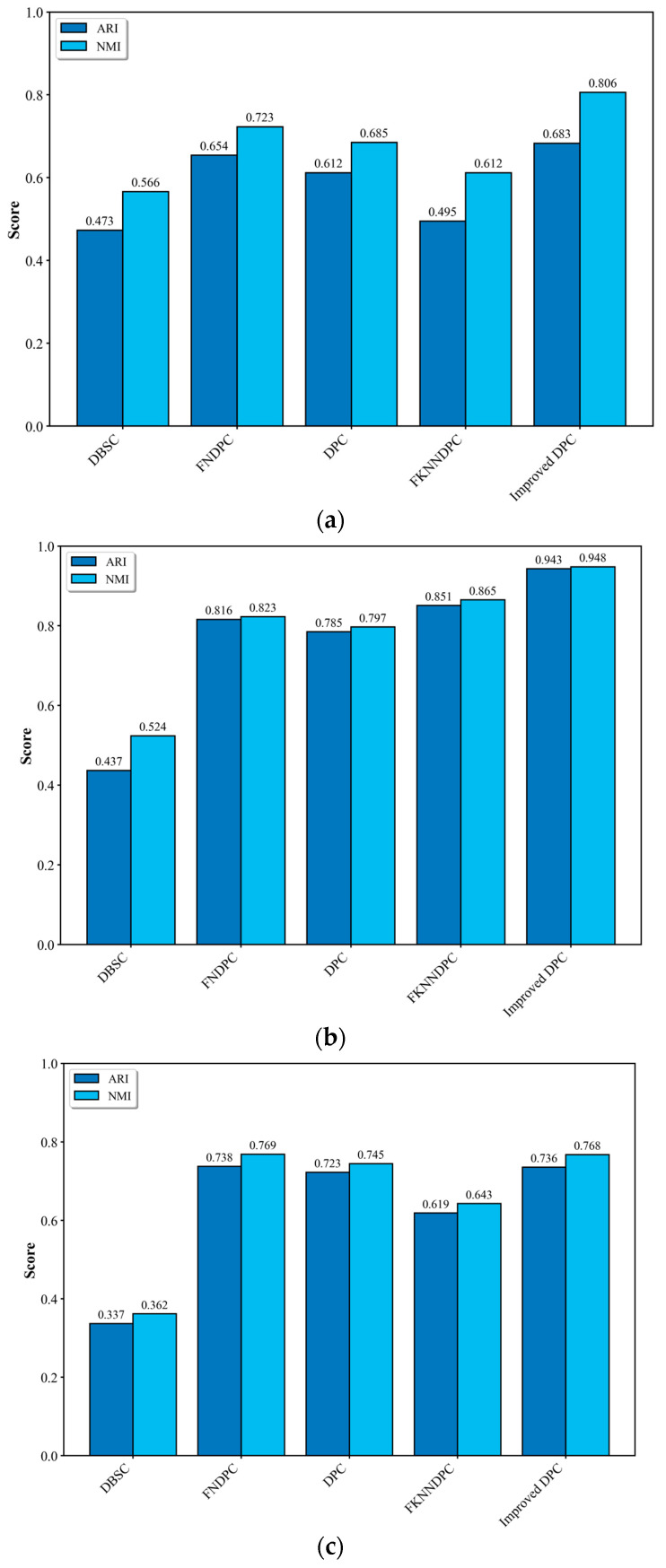
Experimental comparison of various clustering algorithms on UCI dataset. (**a**) Segmentation dataset, (**b**) Dermatology dataset, and (**c**) Ecoli dataset.

In order to verify the accuracy and reliability, the improved DPC algorithm is compared with the DBSC (Density-Based Spatial Clustering) algorithm [45], FNDPC (fuzzy neighborhood Density Peak Clustering) algorithm [46], DPC algorithm [37], and FKNNDPC (Fuzzy K-Nearest Neighbors Density Peak Clustering) algorithm [47] using UCI datasets [48]. The evaluation experiments across multiple datasets of UCI, including Segmentation, Dermatology, and Ecoli, have demonstrated that the improve DPC consistently achieves superior performance with the averaged ARI = 0.787 and NMI = 0.841 (Figure 7).

### 4.5. Comparative Test for Sleep Posture Classification

**Figure 8:**
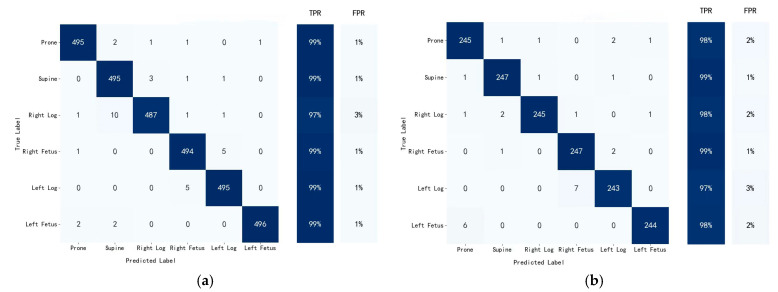
Confusion matrix of PCSN for training and test sets. (**a**) Classification results of training set, (**b**) classification results of test set.

To validate the performance of the PCSN model, two-thirds of the sampling data were used for training (4500 × 2/3=3000) and the remaining one-third for testing (4500 × 1/3=1500). The confusion matrix of the PCSN model in classifying the six sleep postures revealed high accuracy across all categories (Figure 8). The model demonstrated a remarkable ability to correctly identify the supine posture, achieving accuracies of 99% and 98.8% on the training and test datasets, respectively. The right fetus posture was also classified with high precision, with an accuracy of 98.8% for both the training and test datasets. Although the right log and left log postures had the lowest accuracy rates, their accuracies were still above 97%. Overall, the PCSN model exhibited robust performance in classifying sleep postures, achieving high accuracy on both the training and test datasets. The model’s ability to maintain high consistency in performance (average accuracy = 98.67% for training and 98.17% for testing) across different sleep postures suggests that it is not only effective in learning from the training data but also possesses good generalization properties.

**Figure 9:**
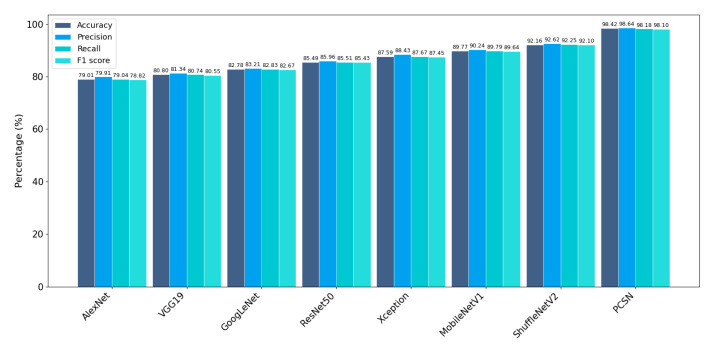
Comparison of PCSN with state-of-the-art deep learning models for sleep posture classification.

In addition, the PCSN model was compared with several classic deep learning models, including AlexNet, VGG19, GoogLeNet, ResNet50, Xception, MobileNetV1, and ShuffleNetV2, using accuracy, precision, recall, and F1 score as metrics for sleep posture classification. The comparative results (Figure 9) show that the PCSN model, which integrates both spatial and temporal features, achieved the highest accuracy (98.42%), precision (98.64%), recall (98.18%), and F1 score (98.10%). These results significantly outperform various state-of-the-art models.

To further evaluate whether the improvement is statistically significant, we have performed paired *t*-tests comparing the proposed PCSN model with state-of-the-art deep learning models mentioned above. The results demonstrate that the *p*-values are consistently below 0.001, providing strong evidence that the performance enhancements of the PCSN model are statistically significant.

## 5. Discussion

In this study, we developed a non-contact sleep posture detection system comprising eight optimally placed accelerometers. This hardware configuration significantly reduces the cost and complexity compared to uniformly distributed sensor array methods [23,24,25,26]. Additionally, we integrated spatial and temporal characteristics into the deep learning model with improved DPC algorithm. The classification results show that the PCSN model achieves an average accuracy of 98.42% for six sleep postures.

### 5.1. PCSN Performance Evaluation

**Figure 10:**
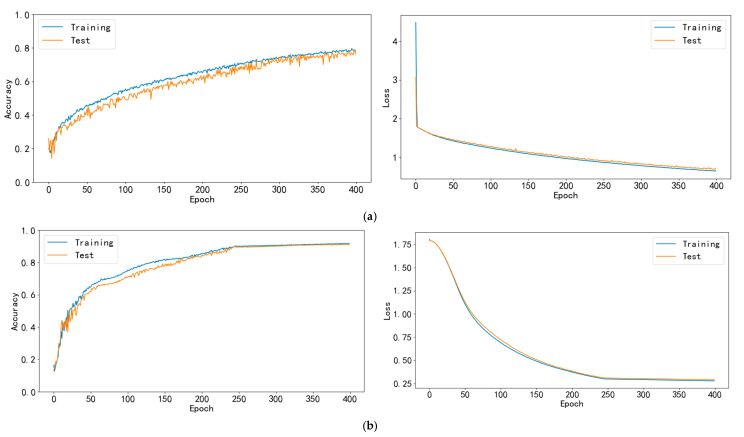

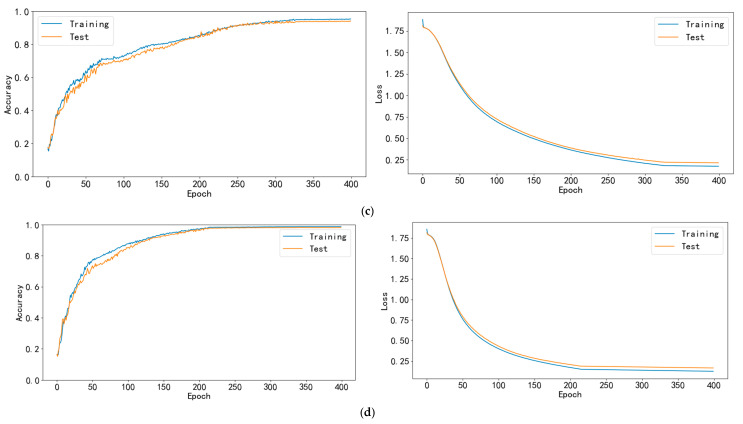
Accuracy and loss value curves of model training and testing sets. (**a**) CNN model, (**b**) CNN + LSTM model, (**c**) CNN + Bi-LSTM model, and (**d**) PCSN (Parallel Convolutional Spatiotemporal Network).

The accuracy and loss values of PCSN model were compared with those of the CNN model, the CNN + LSTM model, and the CNN + Bi-LSTM model for both the training and the test sets (Figure 10).

Regarding the training and test set accuracies (first column of Figure 10), the CNN model demonstrated moderate learning capability, with a noticeable gap between training and test accuracies that remained unstable even after 400 epochs. However, integrating LSTM with CNN significantly enhanced the model’s performance, achieving accuracies of 91.60% and 91.00% for training and test sets after 250 epochs, respectively. Further improvement was observed when Bi-LSTM was added to the CNN framework, resulting in higher training and test accuracies of 95.33% and 94.13% after 350 epochs, respectively. The primary disadvantage of the Bi-LSTM + CNN model was its relatively slow convergence rate. The PCSN model achieved the highest accuracy among all models, with accuracies of 98.67% and 98.17% for training and test sets after only 220 epochs, respectively. These results highlight the importance of considering both spatial and temporal dynamics in model design, particularly for tasks involving sequence prediction.

For the loss function, which provides a quantitative measure of the models’ learning progress and generalization ability (second column of Figure 10), the CNN model demonstrated a sharp decrease in both training and test losses but failed to reach a stable state even after 400 epochs. In contrast, the CNN + LSTM model showed both training and test losses converged towards a relatively small value (0.3 after 250 epochs). The CNN + Bi-LSTM model stabilized at even smaller values after 350 epochs, with loss functions for both training and test data reaching 0.18 and 0.22, respectively. After 220 epochs, the PCSN model demonstrated the smallest loss value, 0.15 and 0.19 for training and test sets, respectively, indicating its exceptional learning and generalization performance.

### 5.2. Comparison with Non-Contact Sleep Posture Detection Methods

The comparative analysis presented in Table 3 evaluates the performance of various non-contact sleep posture detection methods, highlighting the effectiveness of different sensor types and algorithms in accurately classifying sleep postures.

Notably, the pressure mat [29] utilizing AdaBoost and SVM achieved an impressive average accuracy of 99.9%, showcasing the potential of deep learning in this domain. However, such high accuracy comes at the cost of increased computational complexity and resource requirements. Our system distinguishes itself with a remarkable average accuracy of 98.42%. This high accuracy is attributed to two key factors: (1) the optimal sensor placement ensures effective capture of movement without the need for large arrays. This not only reduces the cost but also simplifies the complexity of the system, making it more accessible and easier to implement; (2) the combination of both temporal and spatial information by the PCSN model provides a more comprehensive understanding of sleep postures.

Although various methods can achieve high accuracy in sleep posture detection, our system offers a balanced approach. It maximizes performance while minimizing complexity and cost, making it highly practical for real-world scenarios where ease of use, cost, and reliability are paramount.

### 5.3. Limitation

While this study has undeniably achieved significant results in the field of sleep posture detection, it is essential to acknowledge several limitations that should be considered for future improvements and research directions.

Firstly, the definition of sleep posture categories used in this study is somewhat limited. The posture changes of bed-dependent patients in real-life scenarios are often more complex and diverse than the categories we have defined. In clinical and everyday settings, patients may exhibit a wide range of postures that are not included in our current classification system. However, our system has proved capable of classifying the six typical sleep postures mentioned in the previous literature [18].

Secondly, there was a lack of testing on users of different ages, such as elderly populations who might have different sleep patterns and postures due to age-related physical changes. Additionally, our study did not include individuals with varying body mass indices and heights, which could also influence sleep posture and the effectiveness of our detection system based on the position of the sensors. Including a more diverse range of participants in future studies would help to ensure that our system is robust and applicable across different demographic groups.

Thirdly, the experiments were conducted in a controlled laboratory setting, which may not fully replicate the complexities of real-world environments. The experiments were conducted on a single standard mattress with a thickness of 4.5 cm, which allowed for optimal placement of sensors. However, this controlled setting may not fully represent real-world conditions. The following real-world variables could impact system performance: (1) Different mattress materials, such as memory foam, innerspring, or latex, have varying degrees of firmness and elasticity. These differences can alter how pressure is distributed across the surface of the mattress. (2) The size of the mattress can influence the overall surface area available for pressure distribution. A larger mattress may provide more space for even distribution, whereas a smaller one might concentrate pressure in certain areas. (3) Shared beds introduce complex interference patterns that could have a significant impact on classification accuracy. Each individual’s body movements, such as rolling over, shifting positions, or getting out of bed, generate vibrations that can interfere with each other. These vibrations can create overlapping and interacting patterns that make it more challenging to accurately classify and distinguish individual movements or events. 

Additionally, the relatively short detection period used in our study might not capture the full range of sleep posture changes that occur over a night sleep [52,53,54,55]. In the future, it would be beneficial to apply the system to real-world scenarios, such as overnight sleep posture detection, to better understand its performance over extended periods and in more natural settings.

Despite the above limitation, our cost-effective system has made a significant progress towards addressing the challenges by classifying six typical sleep postures that are frequently listed in the existing literature [23,32,50]. Ultimately, the smart mattress presents a highly effective solution for the detection of sleep postures, which has the potential to significantly enhance health outcomes and elevate the quality of life for individuals across various groups.

The experiments were conducted on a single mattress, which allowed for optimal placement of sensors. However, further research is needed to determine the optimal sensor placement for larger mattresses, such as queen or king size. Additionally, while the current trials were conducted with a single person, further tests are required to assess the effectiveness of this system when two people share the same mattress. Top-of-the-range mattresses often utilize thick mattress toppers. Further research is required to determine if placing accelerometers under these thicker layers can also effectively detect sleeping postures.

## 6. Conclusions

In this study, we developed a non-contact sleep posture detection system using eight optimally deployed triaxial accelerometers, strategically positioned to cover the most sensitive areas of a sleeping person. An improved DPC algorithm, incorporating the K-nearest neighbor mechanism, was utilized to enhance the robustness of the similarity metric. Additionally, a PCSN deep learning model was employed to integrate temporal and spatial information, achieving high-precision classification of sleep postures. The system achieved a classification accuracy of 98.42%. Moreover, the cost of the sensing unit is less than USD 8, with each ADXL345 accelerometer costing less than USD 1. Thus, the findings provide a cost-effective and reliable solution for sleep posture detection.

## Figures and Tables

**Table 1:** Demographic information of all participants reported as mean ± standard deviation.

Participants	Age (Years)	Height (cm)	Body Mass (kg)
Male (15)	23.2 ± 1.6	176.7 ± 4.8	70.4 ± 8.1
Female (15)	22.3 ± 1.4	164.6 ± 1.8	53.1 ± 5.2
Overall (30)	22.7 ± 1.6	170.7 ± 7.1	61.8 ± 11.1

**Table 2:** Quantitative metrics of proposed system.

	System Accuracy (°)	Spatial Domain Resolution (°)	Acceleration Domain Resolution (g)
X Axis	0.18	0.25	0.06
Y Axis	0.16	0.24	0.07
Z Axis	0.17	0.21	0.06

**Table 3:** Comparison with some of non-contact sleep posture detection methods in literatures.

Name	Sensor Type	Configuration	Algorithm	Categories	Accuracy
[12]	accelerometer	8	Rule-based	4	92%
[23]	pressure sensor	64 × 32 = 2048	DLP	6	97.5%
[24]	flexible sensor	2 × 2 = 4	SVM	4	96%
[25]	pressure sensor	20 × 11 = 220	CNN, SVM	4	96.987%
[26]	flexible sensor	4 × 11 = 44	LSTM	4	97.97%
[29]	pressure sensor	11 × 24 = 264	AdaBoost, SVM	3	99.9%
[32]	pressure sensor	64 × 128 = 8192	BEMD	6	91.21%
[49]	pressure sensor	32 × 32 = 1024	ConcatNet	4	95.56%
[50]	piezoelectric sensor	4 × 8 = 32	S3CNN	3	93.02%
[51]	flexible sensor	32 × 32 = 1024	ResNet	6	95.08%
**(Ours)**	**accelerometer**	**8**	**PCSN**	**6**	**98.** **42** **%**

DLP: Deep Learning Position, SVM: Support Vector Machine, CNN: Convolution Neural Network, LSTM: Long Short-Term Memory, S3CNN: Sparse Sensor-based Spatiotemporal Convolution Neural Network, BEMD: Body-Earth Mover’s Distance.

## Data Availability

The data presented in this study are available on request from the corresponding author.

## Abbreviations

The following abbreviations are used in this manuscript:

DLP	Deep Learning Position
SVM	Support Vector Machine
CNN	Convolution Neural Network
LSTM	Long Short-Term Memory
S3CNN	Sparse Sensor-based Spatiotemporal Convolution Neural Network
BEMD	Body-Earth Mover’s Distance
PCSN	Parallel Convolutional Spatiotemporal Network
DBSC	Density-Based Spatial Clustering
FNDPC	fuzzy neighborhood Density Peak Clustering
DPC	Density Peak Clustering
FKNNDPC	Fuzzy K-Nearest Neighbors Density Peak Clustering

## Appendix A

The pseudocode of the algorithm is included in Algorithm A1.

**Algorithm A1** Pseudocode of improved DPC algorithm	
0	K_get_Final_Centers(X,ρ,δ)
1	$\mathrm{Input}:\;\mathrm{Datasets},\;X=\left\{x_{1},x_{2},\ldots ,x_{n}\right\}$, Local density: ρ, Repulsion Value: δ
2	OutPut: Cluster Centers Set: Final_Centers
3	begin
4	for α ← 1 to n do
5	${\gamma \;}_{\alpha }$$\leftarrow \;\text{cal\_}(\rho_{\alpha },\delta_{\alpha }$)
6	end for
7	$\bar{\rho \;}$ ← avg(ρ)
8	$\bar{\delta \;}$ ← avg(δ)
9	$\;\mathrm{centers}\;\leftarrow \;\text{get\_centers}(\bar{\rho },\bar{\delta }$)
10	quick_sort(centers,γ,‘desc’)
11	for α ← 1 to |centers| do
12	$g_{\alpha }\;$ $\leftarrow \;\gamma_{\alpha }-\gamma_{\alpha -1}$
13	end for
14	$\bar{g}$ ← avg(g)
15	for α ← |centers| do 1 do
16	$\mathrm{if}\;g_{\alpha }>\bar{g}$ then
17	$\text{final\_centers}\;\leftarrow \;\text{final\_centers}\;\cup \;x_{\alpha }$
18	end for
19	$\mathrm{if}\;g_{\alpha }\leq \bar{g}$ then
20	break
21	end if
22	end for
23	return final_centers
24	end
